# New Hope for Retinoblastoma Patients

**Published:** 2010-07

**Authors:** Khalil Ghasemi Falavarjani, Masood Naseripour

**Affiliations:** Eye Research Center, Iran University of Medical Sciences, Tehran, Iran

Systemic chemotherapy regimens and focal treatment modalities such as radioactive plaque therapy have dramatically improved the outcomes of treatment for retinoblastoma such that patient survival with modern monitoring and treatment now exceeds 95%. Although the vast majority of patients respond adequately to current therapeutic strategies, treatment for a subset of patients with advanced retinoblastoma remains challenging. Consider an infant with advanced bilateral retinoblastoma, group D international classification (diffuse vitreous and subretinal seedings of the tumor) in one eye and group E (anterior segment invasion of the tumor) in the fellow eye; treatment options for such a patient are limited to repetitive courses of systemic and periocular chemotherapy, and/or external beam radiation therapy. These modalities are usually associated with significant adverse effects, including bone marrow suppression, hearing loss, systemic infections and increased risk of secondary cancers. In another small group of patients, intraocular tumors may regrow despite aggressive local and systemic treatment and no other choice remains but enucleation.

In a novel approach, Abramson et al presented their initial experience with superselective ophthalmic artery delivery of chemotherapy (chemosurgery) for children with advanced retinoblastoma in eyes scheduled for enucleation. They cannulated the ophthalmic artery via a femoral artery approach using microcatheters while the children were under general anesthesia and receiving anticoagulation. A balloon in the catheter was inflated, occluding the carotid artery and a chemotherapeutic agent (melphalan) was infused proximal to the occlusion. After treatment, dramatic regression of tumors, vitreous seeds and subretinal seeds were seen in all eyes while no major systemic or local side effect occurred. Among the 9 cases treated with this technique, 7 eyes (77.7%) destined to be enucleated were salvaged. Using electroretinography, the investigators showed that retinal function may persist and even recover following this technique. In a complementary article, they reported 4 patients with advanced bilateral disease (groups C–E) in whom initial management was bilateral superselective ophthalmic artery chemotherapy performed during the same session. All tumors and vitreous seeds in all subjects showed significant reduction in size within 3 weeks of treatment and no eye required radiation or enucleation.

Direct infusion of chemotherapeutic agents into the ophthalmic artery seems to be effective and safe both for the patient and the eye. Since chemosurgery does not require ports, transfusions, hospitalizations, and does not incur neutropenia or infections, the cost of therapy is much reduced. A cost analysis of intraarterial chemosurgery has estimated the cost of this method to be approximately half that of systemic chemotherapy. These promising initial results suggest that this approach may pave the way for treatment of advanced intraocular retinoblastoma while avoiding the use of multiagent systemic chemotherapy, enucleation, and/or radiation.
